# Tumor Necrosis Factor-α Mediates Lung Injury in the Early Phase of Endotoxemia

**DOI:** 10.3390/ph15030287

**Published:** 2022-02-25

**Authors:** Kung-Yen Chen, Chao-Yuan Chang, Hao-Jen Hsu, Hung-Jen Shih, I-Tao Huang, Hemal H. Patel, Chun-Jen Huang

**Affiliations:** 1 Department of Anesthesiology, Wan Fang Hospital, Taipei Medical University, Taipei 116, Taiwan; 95352@w.tmu.edu.tw; 2 Department of Anesthesiology, School of Medicine, College of Medicine, Taipei Medical University, Taipei 110, Taiwan; yuanc669@gmail.com; 3 Integrative Research Center for Critical Care, Wan Fang Hospital, Taipei Medical University, Taipei 116, Taiwan; 4 Department of Medical Research, Wan Fang Hospital, Taipei Medical University, Taipei 116, Taiwan; 5 Department of Life Sciences, College of Medicine, Tzu Chi University, Hualien 970, Taiwan; hjhsu32@mail.tcu.edu.tw; 6 Department of Urology, Changhua Christian Hospital, Changhua 500, Taiwan; jasta1206@gmail.com; 7 Department of Recreation and Holistic Wellness, MinDao University, Changhua 523, Taiwan; 8 Department of Urology, School of Medicine, College of Medicine, Taipei Medical University, Taipei 110, Taiwan; 9 Emergency Department, Redcliffe Hospital, Redcliffe, QLD 4020, Australia; itao.huang@uqconnect.edu.au; 10 School of Public Health, Faculty of Medicine, University of Queensland, Brisbane, QLD 4006, Australia; 11 VA San Diego Healthcare System, San Diego, CA 92161, USA; 12 Department of Anesthesiology, University of California, San Diego, CA 92161, USA; 13 Graduate Institute of Clinical Medicine, College of Medicine, Taipei Medical University, Taipei 110, Taiwan

**Keywords:** lung injury, endotoxin, cytokines, cytokine receptors, peptide, mice

## Abstract

Endotoxemia induces lung injury. We assessed the therapeutic efficacy between triple cytokine (tumor necrosis factor-α [TNF-α], interleukin-1β [IL-1β], and IL-6) inhibition (mediated by KCF18 peptide) and single cytokine (TNF-α) inhibition (mediated by SEM18 peptide) on alleviating lung injury in the early phase of endotoxemia. Mice receiving endotoxin (Endo group), endotoxin plus KCF18 (EKCF group), or endotoxin plus SEM18 (ESEM) were monitored and euthanized at 24 h after endotoxin. Our data demonstrated altered lung function (decreases in tidal volume, minute ventilation, and dynamic compliance; and by contrast, increases in airway resistance and end expiration work) and histology (increases in injury scores, leukocyte infiltration, vascular permeability, and tissue water content) in the Endo group with significant protection observed in the EKCF and ESEM groups (all *p* < 0.05). Levels of inflammation (macrophage activation and cytokine upregulations), oxidation (lipid peroxidation), necroptosis, pyroptosis, and apoptosis in EKCF and ESEM groups were comparable and all were significantly lower than in the Endo group (all *p* < 0.05). These data demonstrate that single cytokine TNF-α inhibition can achieve therapeutic effects similar to triple cytokines TNF-α, IL-1β, and IL-6 inhibition on alleviating endotoxin-induced lung injury, indicating that TNF-α is the major cytokine in mediating lung injury in the early phase of endotoxemia.

## 1. Introduction

The critical role of inflammatory cytokines in mediating systemic inflammation and the development of organ dysfunction and injury (e.g., the lungs) in sepsis are established [1,2]. In the early phase of sepsis, inflammatory cytokines, including tumor necrosis factor-α (TNF-α), interleukin-1β (IL-1β), and interleukin-6 (IL-6) are manufactured from infection-triggered immune cells (e.g., macrophages) and stromal cells (e.g., epithelial cells, endothelial cells, and fibroblasts) through activation of toll-like receptor (TLR) and nuclear factor-κB (NF-κB), and then extricated into systemic circulation [3,4]. Clinical data highlight the correlation between the concentrations of TNF-α, IL-1β, and IL-6 and the severity of organ dysfunction/injury in septic patients [5,6]. Thus, these cytokines are identified as markers of systemic inflammation and represent potential therapeutic targets against sepsis [7].

With its extrication into circulation in the early phase of sepsis, TNF-α can in turn escalate the systemic inflammatory response [8]. The mechanisms mainly involve activation of NF-κB through its binding with the cognate receptors, TNF receptor 1 (TNFR1) and TNFR2, and subsequently enhancing production of inflammatory cytokines (including TNF-α *per se,* IL-1β, and IL-6) [8]. TNF-α is considered a primary modulator of inflammation [8]. Upon binding with TNFR1, TNF-α can activate the cell death processes of necroptosis and apoptosis [9,10,11]. Moreover, necroptosis can promote pyroptosis, through activating the inflammasome nucleotide-binding oligomerization domain-like receptor protein 3 (NLRP3) [12]. With its effects on escalating inflammation and activating the cell death processes of necroptosis, pyroptosis, and apoptosis, the major role of TNF-α in mediating sepsis-induced vital organ injury (e.g., the lungs) is established [8,9,10,11,12].

IL-1β, bearing resemblance to TNF-α, can also escalate the systemic inflammatory response in septic patients [13]. The mechanisms involve its effect on activating NF-κB through its binding with the cognate receptor IL-1 receptor (IL-1R), subsequent recruitment of MyD88, and the resultant upregulation of inflammatory cytokines (e.g., IL-6, etc.) and chemokines [13,14]. Moreover, with NLRP3 inflammasome and caspase-1 activation, pyroptosis can lead to exteriorization of cellular IL-1β and further enhance the systemic inflammatory response in sepsis [15]. As TNF-α can activate NF-κB and pyroptosis [8,12], TNF-α can modulate the transcription and expression of IL-1β implicating it as a more proximal effector.

Notably, TNF-α and IL-1β can integrate to upregulate IL-6 [8,13,16]. IL-6 is a cytokine with multiple functions [17]. IL-6 can stimulate synthesis of acute phase proteins and modulate activation of immune cells during sepsis [17]. Upon binding with its cognate receptor, IL-6 receptor (IL-6R), and subsequent activation of the downstream Akt pathway, IL-6 can induce vascular endothelial breakdown and aggravate organ injury (e.g., the lungs) during sepsis [18].

These studies collectively highlighted the important roles of TNF-α, IL-1β and IL-6 in mediating organ injury (e.g., the lungs) in sepsis. Our study engaged a widely used Gram (-) endotoxin intraperitoneal (i.p.) administration model to engender endotoxemia (namely monomicrobial sepsis) in mice [19]. This study also investigated the impact of therapy on modulating crucial mechanisms mediating endotoxemia-induced lung injury, including inflammation, oxidation [20], and cell death processes of necroptosis, pyroptosis, and apoptosis. KCF18 peptide, possessing structural similarity to the binding domains of TNF-α, IL-1β, and IL-6 to TNFR1, IL-1R, and IL-6R, served as a binding decoy to achieve simultaneous blockings of TNF-α, IL-1β, and IL-6 [21,22]. SEM18 peptide, possessing the structural similarity to the binding domain of TNF-α to TNFR1, served as a binding decoy to achieve TNF-α blockade [21,23]. Considering the primary role of TNF-α in modulating the expression of IL-1β and IL-6, we hypothesized that TNF-α may have a more immediate role in systemic inflammation and organ injury (e.g., the lungs) in endotoxemia. In line with this notion, we further hypothesized that blocking the three early response cytokines TNF-α, IL-1β, and IL-6 (mediated by the KCF18 peptide) and blocking only TNF-α (mediated by the SEM18 peptide) may achieve similar effects on mitigating endotoxemia-induced systemic inflammation and organ injury.

## 2. Results

### 2.1. Effects of KCF18 and SEM18 on Inhibiting Endotoxemia-Induced Cytokine Upregulations in Plasma

Adult male mice were randomly allocated to receive normal saline, normal saline plus the KCF18 peptide, normal saline plus the SEM18 peptide, endotoxin, endotoxin plus the KCF18 peptide, or endotoxin plus the SEM18 peptide (identified as the Sham, the KCF, the SEM, the Endo, the EKCF, and the ESEM group, respectively). Our data demonstrated that all mice survived the experiments and the survival rates at 24 h after normal saline or endotoxin treatment in the Sham, KCF, SEM, Endo, EKCF, and ESEM groups were all 100%.

**Figure 1:**
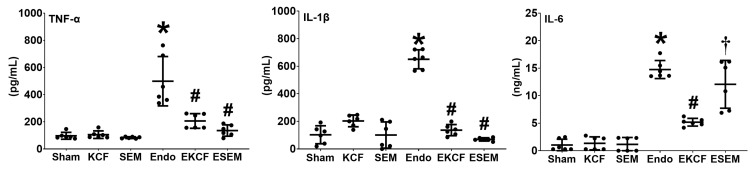
Systemic inflammation status. Plasma concentrations of tumor necrosis factor-α (TNF-α), interleukin-1β (IL-1β), and interleukin-6 (IL-6) were measured at 24 h after endotoxin or normal saline administration, using enzyme-linked immunosorbent assay. Sham: the normal saline group. KCF: the normal saline plus the KCF18 peptide group. SEM: the normal saline plus the SEM18 peptide group. Endo: the endotoxin group; EKCF: the endotoxin plus the KCF18 peptide group. ESEM: the endotoxin plus the SEM18 peptide group. Data were obtained from 6 mice in each group and presented as the mean ± standard deviation. * *p* < 0.05, versus the Sham group. # *p* < 0.05, versus the Endo group. † *p* < 0.05, the ESEM group versus the EKCF group.

Figure 1 illustrates plasma cytokine concentrations measured at 24 h post-treatment using enzyme-linked immunosorbent assay (ELISA). The plasma concentrations of TNF-α in Sham, KCF, and SEM groups were low. In contrast, the plasma concentration of TNF-α in the Endo group was significantly higher than that in the Sham group (*p* < 0.001). Notably, the plasma concentrations of TNF-α in the EKCF and the ESEM groups were comparable (*p* = 0.662) and both were significantly lower than the Endo group (both *p* < 0.001). The comparisons of the IL-1β data paralleled that of the TNF-α data. These data demonstrate the comparable and significant effects of the KCF18 and SEM18 peptides on inhibiting endotoxemia-induced upregulation of TNF-α and IL-1β in mouse plasma. The comparison of the IL-6 data also paralleled that of the TNF-α data, except that the plasma IL-6 concentrations in the ESEM and the Endo groups were not significantly different (*p* = 0.254) and both were significantly higher than that of the EKCF group (both *p* < 0.001). These data demonstrate the differential effects of the KCF18 and SEM18 peptides on inhibiting endotoxemia-induced upregulation of IL-6 in mouse plasma.

### 2.2. Effects of KCF18 and SEM18 on Inhibiting Endotoxemia-Induced Cytokine-Receptor Binding in Lungs

**Figure 2:**
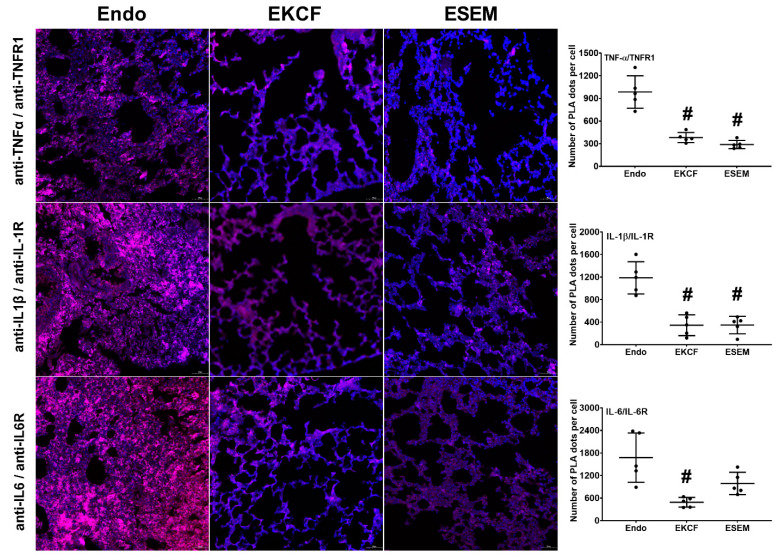
Cytokine-receptor binding status. Representative microscopic images of immunofluorescence staining of proximity ligation assay (PLA) and PLA signal intensities for measuring bindings of tumor necrosis factor-α (TNFα), interleukin-1β (IL-1β), and interleukin-6 (IL-6) to the cognate receptor TNF receptor 1 (TNFR1), IL-1 receptor (IL-1R), and IL-6 receptor (IL-6R) in lung tissues in mice that were measured at 24 h after endotoxin administration. Red dots: (+) PLA signal, indicating (+) protein–protein interactions between TNF-α/TNFR1, IL-1β/IL-1R, and IL-6/IL-6R. Blue dots: 4′,6-diamidino-2-phenylindole stain, indicating a cell nucleus in lung tissues. Endo: the endotoxin group. EKCF: the endotoxin plus the KCF18 peptide group. ESEM: the endotoxin plus the SEM18 peptide group. Data were obtained from five mice in each group and presented as the mean ± standard deviation. # *p* < 0.05, versus the Endo group.

Figure 2 illustrates endotoxemia-induced cytokine-receptor binding assay measured at 24 h after endotoxin administration using the proximity ligation assay (PLA). The respective pulmonary PLA signal intensity of TNF-α/TNFR1 (i.e., the indicator of TNF-α/TNFR1 binding) in the EKCF group and the ESEM group was approximately 38.7% and 29.3% of that in the Endo group. The analysis revealed that the TNF-α/TNFR1 PLA signal intensities in the EKCF and ESEM groups were comparable (*p* = 0.531) and both were significantly lower than the Endo group (both *p* < 0.001). These data demonstrate the comparable and significant effects of the KCF18 and SEM18 peptides on inhibiting endotoxemia-induced TNF-α/TNFR1 binding in lung tissue.

The respective pulmonary IL-1β/IL-1R PLA signal intensity in the EKCF group and the ESEM group was approximately 29.0% and 29.4% of that in the Endo group. The analysis revealed that the IL-1β/IL-1R PLA signal intensities in the EKCF and ESEM groups were comparable (*p* = 0.999) and both were significantly lower than the Endo group (both *p* < 0.001). Similarly, these data demonstrate the comparable and significant effects of the KCF18 and SEM18 peptides on inhibiting endotoxemia-induced IL-1β/IL-1R binding in lung tissue.

The respective pulmonary IL-6/IL-6R PLA signal intensities in the EKCF group and the ESEM group were approximately 29.2% and 59.0% of that in the Endo group. The analysis revealed that the IL-6/IL-6R PLA signal intensities in the EKCF and ESEM groups were not significantly different (*p* = 0.190), indicating comparable effects of the KCF18 peptide and the SEM18 peptide on inhibiting endotoxin-induced IL-6/IL-6R binding in lung tissues. The analysis also revealed that the IL-6/IL-6R PLA signal intensity in the EKCF group was significantly lower than the Endo group (*p* = 0.002), indicating the KCF18 peptide can significantly inhibit endotoxin-induced IL-6/IL-6R binding in lung tissues. However, though the IL-6/IL-6R PLA signal intensity in the ESEM group was lower than the Endo group, analysis revealed that the difference between these two groups did not reach statistical significance (*p* = 0.059). These data indicated that the SEM18 peptide did not exert significant effect on inhibiting endotoxemia-induced IL-6/IL-6R binding in mouse lung tissue.

### 2.3. Effects of KCF18 and SEM18 on Alleviating Endotoxemia-Induced Lung Function Alterations

**Figure 3:**
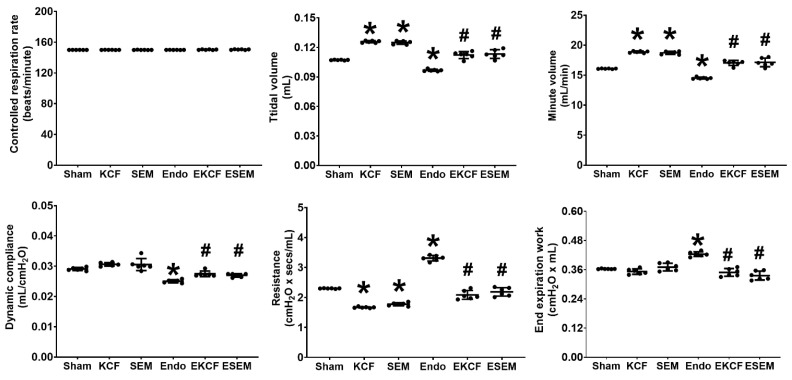
Lung function status. Lung function parameters, including controlled respiration rate, tidal volume, minute volume, dynamic compliance, resistance, and end expiration work were measured at 24 h after endotoxin or normal saline administration. Sham: the normal saline group. KCF: the normal saline plus the KCF18 peptide group. SEM: the normal saline plus the SEM18 peptide group. Endo: the endotoxin group. EKCF: the endotoxin plus the KCF18 peptide group. ESEM: the endotoxin plus the SEM18 peptide group. Data were obtained from six mice in each group and presented as the mean ± standard deviation. * *p* < 0.05, versus the Sham group. # *p* < 0.05, versus the Endo group.

With controlled mechanical ventilation (ventilation rate of 150 breaths/min and a tidal volume of 0.2 mL), lung function parameters, including respiration rate, tidal volume, minute volume, dynamic compliance, resistance, and end expiration work, were measured at 24 h after normal saline or endotoxin administration. Figure 3 illustrates lung function results. As all mice were under controlled mechanical ventilation, the respiratory rates of all six groups were comparable. Though the tidal volume was set at 0.2 mL, the measured tidal volumes of the KCF and the SEM groups were significantly higher than that of the Sham group (both *p* < 0.001), indicating the effects the KCF18 peptide and the SEM18 peptide on increasing tidal volume in mice with mechanical ventilation. Of note, the measured tidal volume of the Endo group was significantly lower than that of the Sham group (*p* < 0.001). Moreover, the measured tidal volumes of the EKCF and the ESEM groups were comparable (*p* = 0.977) and both were significantly higher than that of the Endo group (both *p* < 0.001). These data demonstrate the comparable effects of the KCF18 and SEM18 peptides on increasing tidal volume in endotoxemia mice with mechanical ventilation. Our data also demonstrate that minute ventilation and dynamic compliance parallel that of the tidal volume data, except that the dynamic compliance of the Sham, the KCF, and the SEM groups were comparable.

The airway resistance of the KCF and the SEM groups were significantly lower than that of the Sham group (both *p* < 0.001), indicating the effects of the KCF18 and SEM18 peptides on decreasing airway resistance in mice with mechanical ventilation. Of note, the airway resistance of the Endo group was significantly higher than that of the Sham group (*p* < 0.001). Moreover, the airway resistance of the EKCF and the ESEM groups were comparable (*p* = 0.451) and both were significantly lower than that of the Endo group (both *p* < 0.001). These data demonstrate the comparable effects of the KCF18 and SEM18 peptides on decreasing airway resistance in endotoxemia mice with mechanical ventilation. Our data also demonstrate that end expiration work parallels that of the airway resistance, except that the end expiration work of the Sham, the KCF, and the SEM groups were comparable.

### 2.4. Effects of KCF18 and SEM18 on Alleviating Endotoxemia-Induced Lung Injury

**Figure 4:**
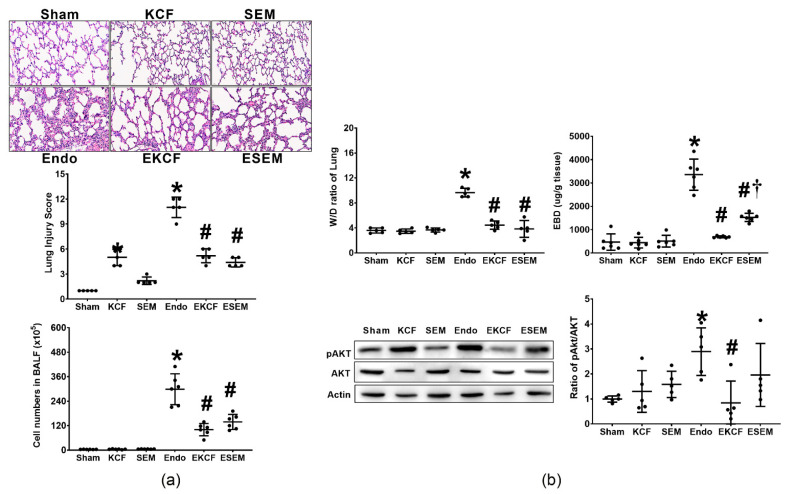
Lung injury status. (**a**) Representative microscopic images of the lung tissues stained with hematoxylin-eosin (200×) and the lung injury scores (data were obtained from five mice in each group). The cell numbers in bronchoalveolar fluid (BALF) (the indicator of leukocyte infiltration; data were obtained from six mice in each group). (**b**) Wet/dry weight (W/D) ratio (the indicator of tissue water content; data were obtained from five mice in each group) and Evans blue dye (EBD) concentrations (the indicator of vascular permeability; data were obtained from six mice in each group). The representative gel images of phosphorylated Akt (pAkt), Akt, and actin (the internal standard), and the relative band density of pAkt/actin in lung tissues. Expression of pAKt, Akt, and actin was assayed using immunoblotting assay (data were obtained from five mice in each group). All assays were measured at 24 h after normal saline or endotoxin administration. Sham: the normal saline group. KCF: the normal saline plus the KCF18 peptide group. SEM: the normal saline plus the SEM18 peptide group. Endo: the endotoxin group. EKCF: the endotoxin plus the KCF18 peptide group. ESEM: the endotoxin plus the SEM18 peptide group. Data were presented as the mean ± standard deviation. * *p* < 0.05, versus the Sham group. # *p* < 0.05, versus the Endo group. † *p* < 0.05, the ESEM group versus the EKCF group.

Lung injury status was determined by evaluating histological characteristics, injury scores, and leukocyte infiltration (measured by cell numbers in bronchoalveolar fluid [BALF]), as well as tissue water content (measured by wet/dry weight [W/D] ratio), vascular permeability (measured by Evans blue dye [EBD] extravasation assay), and Akt expression in lung tissue measured at 24 h after normal saline or endotoxin administration. Figure 4a illustrates histological characteristics, lung injury scores, and cell number in BALF. Our data revealed minimal lung injury characteristics and low lung injury scores in the Sham and the SEM groups. However, histological analysis revealed mild lung injury characteristics in the KCF group and the lung injury score in the KCF group was significantly higher than that in the Sham group (*p* < 0.001), indicating KCF18 peptide induced mild lung injury in mice. Of note, histological analysis revealed significant lung injury characteristics in the Endo group and the lung injury score in the Endo group was significantly higher than that in the Sham group (*p* < 0.001). Moreover, comparing to the Endo group, histological analysis revealed lower levels of lung injury characteristics in both the EKCF and the ESEM groups. The lung injury scores in the EKCF and the ESEM groups were comparable (*p* = 0.599) and both were significantly lower than that of the Endo group (both *p* < 0.001). Of note, the trend of the cell numbers in BALF data paralleled that of the injury score data, except that the cell numbers in BALF in the Sham, the KCF, and the SEM group were low and comparable. These data demonstrate the comparable effects of the KCF18 and SEM18 peptides on alleviating endotoxemia-induced lung injury in mice.

Figure 4b illustrates W/D ratio, EBD concentration, and Akt expression in lung tissue. Of note, the trend of the W/D ratio data paralleled that of the cell number in BALF data. The trend of the EBD concentration data also paralleled that of the cell number in BALF data, except that the EBD concentration in lung tissues in the ESEM group was significantly higher than that in the EKCF group (*p* = 0.003). The expression levels of pAkt in the Sham, the KCF, and the SEM groups were also comparable. As expected, the expression level of pAkt in the Endo group was significantly higher than the Sham group (*p* = 0.003). The expression level of pAkt in the EKCF group was significantly lower than that in the Endo group (*p* < 0.001), whereas the expression levels of pAkt in the ESEM and Endo groups were not significantly different (*p* = 0.287). Moreover, though the expression level of pAkt in the ESEM group was higher than that in the EKCF group, analysis revealed that the difference between these two groups did not reach statistical significance (*p* = 0.124). These data demonstrate the differential effects of the KCF18 and SEM18 peptides on inhibiting endotoxemia-induced vascular permeability increases and upregulation of pAkt in lung tissues in mice.

### 2.5. Effects of KCF18 and SEM18 on Alleviating Endotoxemia-Induced Lung Inflammation and Oxidation

**Figure 5:**
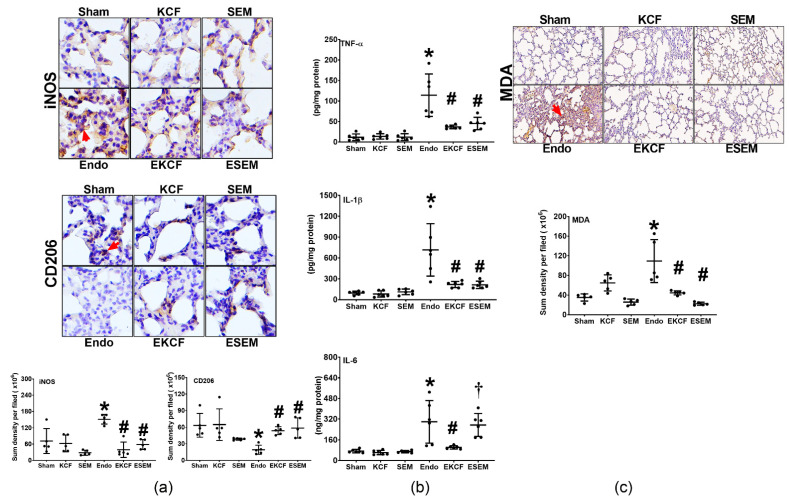
Lung inflammation and oxidation status. (**a**) Representative microscopic images of inducible nitric oxide synthase (iNOS, the indicator of M1 phase polarization; marked by the red arrow, 200×) and CD206 (the indicator of M2 phase polarization; marked by the red arrow, 200×) in lung tissues assayed using immunohistochemistry analysis and the sum of the iNOS and CD206 signal intensities. Data were obtained from five mice in each group. (**b**) The concentrations of tumor necrosis factor-α (TNF-α), interleukin-1β (IL-1β), and interleukin-6 (IL-6) in lung tissues. Data were obtained from six mice in each group. (**c**) The concentrations of malondialdehyde (MDA, the indicator of oxidation) in lung tissues. Data were obtained from five mice in each group. All assays were measured at 24 h after endotoxin or normal saline administration. Sham: the normal saline group. KCF: the normal saline plus the KCF18 peptide group. SEM: the normal saline plus the SEM18 peptide group. Endo: the endotoxin group. EKCF: the endotoxin plus the KCF18 peptide group. ESEM: the endotoxin plus the SEM18 peptide group. Data were presented as the mean ± standard deviation. * *p* < 0.05, versus the Sham group. # *p* < 0.05, versus the Endo group. † *p* < 0.05, the ESEM group versus the EKCF group.

Lung inflammation status was determined by assaying the level of macrophage activation (measured by expression assays of the M1 phase polarization marker, inducible nitric oxide synthase [iNOS] and the M2 phase polarization marker, CD206) and the levels of TNF-α, IL-1β, and IL-6 upregulation in lung tissues. Lung oxidation status was determined by assaying malondialdehyde (MDA) concentrations in lung tissue. Measurements of inflammation and oxidation were conducted at 24 h after normal saline or endotoxin administration. Figure 5a illustrates macrophage activation in lung tissues. Our data demonstrate that the expression levels of iNOS in the Sham, KCF, and SEM groups were low, whereas the expression level of iNOS in the Endo group was significantly higher than that of the Sham group (*p* = 0.001). The expression levels of iNOS in the EKCF and the ESEM groups were comparable (*p* = 0.961) and both were significantly lower than that of the Endo group (both *p* < 0.001). In contrast, the expression levels of CD206 in the Sham, the KCF, and the SEM groups were high and the expression level of CD206 in the Endo group was significantly lower than that in the Sham group (*p* = 0.005). The expression levels of CD206 in the EKCF and the ESEM groups were comparable (*p* = 0.997) and both were significantly higher than that in the Endo group (*p* = 0.039 and 0.014). These data demonstrate the comparable effects of the KCF18 and SEM18 peptides on inhibiting endotoxemia-induced macrophage activation in lung tissues in mice.

Figure 5b illustrates TNF-α, IL-1β, and IL-6 concentrations in lung tissue. Figure 5c illustrates changes in MDA in lung tissue. Of note, the trends of the TNF-α data, the IL-1β data, and the MDA data paralleled that of the iNOS data. These data demonstrate the comparable effects of the KCF18 and SEM18 peptides on inhibiting endotoxemia-induced upregulation of TNF-α and IL-β as well as oxidant stress in lung tissue in mice. The trend of the IL-6 data also paralleled that of the iNOS data, except that the IL-6 level in the ESEM group was significantly higher than that in the EKCF group (*p* = 0.006). These data demonstrate the differential effects of the KCF18 and SEM18 peptides on inhibiting endotoxemia-induced IL-6 upregulation in mouse lung tissue.

### 2.6. Effects of KCF18 and SEM18 on Alleviating Endotoxemia-Induced Lung Necroptosis

**Figure 6:**
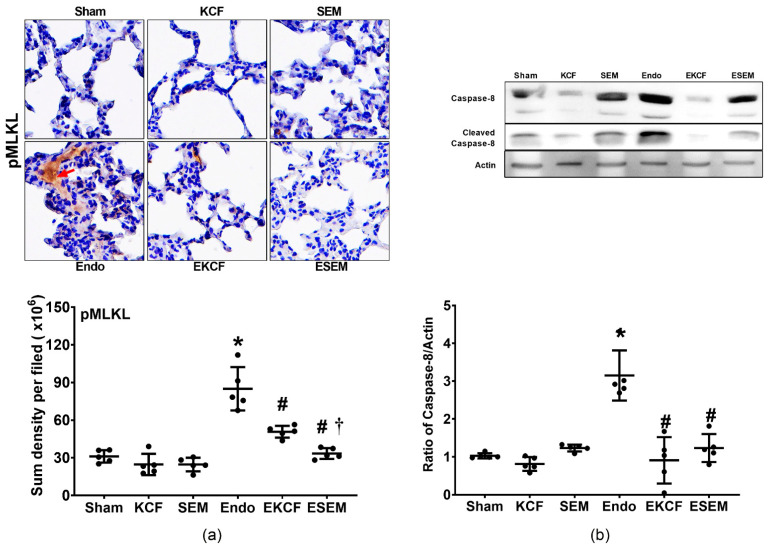
Lung necroptosis status. (**a**) Representative microscopic images of phosphorylated mixed lineage kinase domain-like pseudokinase (pMLKL, the indicator of necroptosis, marked by the red arrow, 200×) in lung tissues assayed using immunohistochemistry analysis and the sum of the pMLKL signal intensity. (**b**) Representative gel images of cleaved caspase-8 and actin (the internal standard) in lung tissues assayed using immunoblotting assay and the relative band density of cleaved caspase-8/actin in lung tissues. All assays were measured at 24 h after endotoxin or normal saline administration. Sham: the normal saline group. KCF: the normal saline plus the KCF18 peptide group. SEM: the normal saline plus the SEM18 peptide group. Endo: the endotoxin group. EKCF: the endotoxin plus the KCF18 peptide group. ESEM: the endotoxin plus the SEM18 peptide group. Data were obtained from five mice in each group and presented as the mean ± standard deviation. * *p* < 0.05, versus the Sham group. # *p* < 0.05, versus the Endo group. † *p* < 0.05, the ESEM group versus the EKCF group.

Lung necroptosis status was determined by assaying expression levels of phosphorylated mixed lineage kinase domain-like pseudokinase (pMLKL) and cleaved caspase-8 in lung tissue at 24 h after normal saline or endotoxin administration. Figure 6a illustrates pMLKL and Figure 6b cleaved caspase-8 in lung tissue. Our data demonstrate that the expression levels of pMLKL and cleaved caspase-8 in the Sham, KCF, and SEM groups were low, whereas the expression level of pMLKL and cleaved caspase-8 in the Endo group were significantly higher than those in the Sham group (both *p* < 0.001). The expression levels of pMLKL and cleaved caspase-8 in the EKCF and ESEM groups were significantly lower than those in the Endo group (all *p* < 0.001). However, the expression level of pMLKL in the EKCF group was significantly higher than that in the ESEM group (*p* = 0.047), whereas the expression levels of cleaved caspase-8 in the EKCF and ESEM groups were not significantly different (*p* = 0.686).

### 2.7. Effects of KCF18 and SEM18 on Alleviating Endotoxemia-Induced Lung Pyroptosis

**Figure 7:**
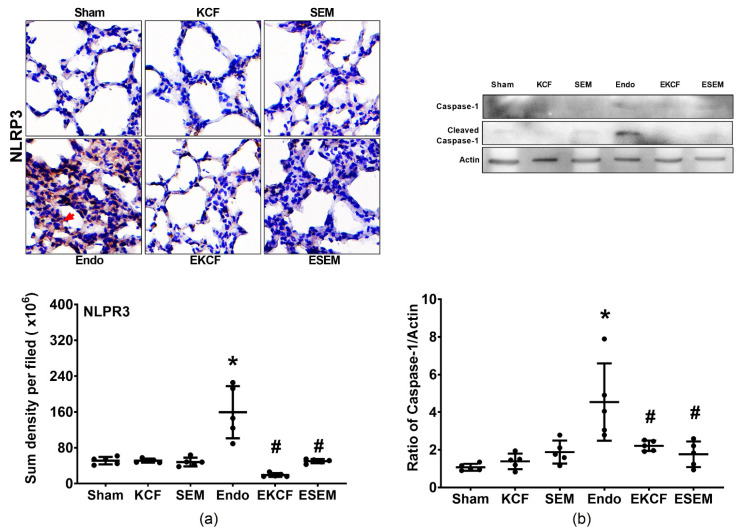
Lung pyroptosis status. (**a**) Representative microscopic images of nucleotide-binding oligomerization domain-like receptor protein 3 (NLRP3, the indicator of pyroptosis, marked by the red arrow, 200×) in lung tissues assayed using immunohistochemistry analysis and the sum of the NLRP3 signal intensity. (**b**) Representative gel images of cleaved caspase-1 and actin (the internal standard) in lung tissues assayed using immunoblotting assay and the relative band density of cleaved caspase-1/actin in lung tissues. All assays were measured at 24 h after endotoxin or normal saline administration. Sham: the normal saline group. KCF: the normal saline plus the KCF18 peptide group. SEM: the normal saline plus the SEM18 peptide group. Endo: the endotoxin group. EKCF: the endotoxin plus the KCF18 peptide group. ESEM: the endotoxin plus the SEM18 peptide group. Data were obtained from 5 mice in each group and presented as the mean ± standard deviation. * *p* < 0.05, versus the Sham group. # *p* < 0.05, versus the Endo group.

Lung pyroptosis status was determined by assaying expression levels of NLRP3 and cleaved caspase-1 in lung tissues at 24 h after normal saline or endotoxin administration. Figure 7 illustrates lung pyroptosis, as measured by assaying expression levels of NLRP3 (Figure 7a) and cleaved caspase-1 (Figure 7b) in lung tissue. Our data demonstrate that the expression levels of NLRP3 and cleaved caspase-1 in the Sham, KCF, and SEM groups were low, whereas the expression level of NLRP3 and cleaved caspase-1 in the Endo group were significantly higher than those in the Sham group (both *p* < 0.001). The expression levels of NLRP3 in the EKCF and ESEM groups were comparable (*p* = 0.372) and both were significantly lower than that in the Endo group (both *p* < 0.001). The expression levels of cleaved caspase-1 in the EKCF and the ESEM group were also comparable (*p* = 0.994) and both were also significantly lower than that in the Endo group (*p* = 0.039 and 0.011). These data demonstrate the comparable effects of the KCF18 and SEM18 peptides on inhibiting endotoxemia-induced pyroptosis in mouse lung tissue.

### 2.8. Effects of KCF18 and SEM18 on Alleviating Endotoxemia-Induced Lung Apoptosis

**Figure 8:**
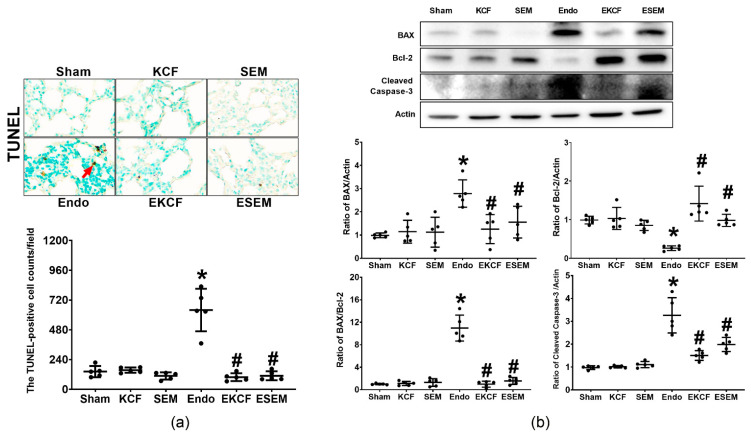
Lung apoptosis status. (**a**) Representative DNA fragmentation microscopic images (the indicator of apoptosis, marked by the red arrow, 200×) in lung tissues assayed using the terminal deoxynucleotidyl transferase dUTP nick end labeling (TUNEL) method and the count of TUNEL-positive cells (0.25 mm^2^). (**b**) Representative gel images of the proapoptotic BAX, the antiapoptotic Bcl-2, proapoptotic cleaved caspase-3, and actin (the internal standard) in lung tissues assayed using immunoblotting assay and the relative band density of BAX/actin, Bcl-2/actin, cleaved caspase-3/actin, and BAX/Bcl-2 ratios in lung tissues. All assays were measured at 24 h after endotoxin or normal saline administration. Sham: the normal saline group. KCF: the normal saline plus the KCF18 peptide group. SEM: the normal saline plus the SEM18 peptide group. Endo: the endotoxin group. EKCF: the endotoxin plus the KCF18 peptide group. ESEM: the endotoxin plus the SEM18 peptide group. Data were obtained from 5 mice in each group and presented as the mean ± standard deviation. * *p* < 0.05, versus the Sham group. # *p* < 0.05, versus the Endo group.

The status of apoptosis in lung cells was determined by assaying DNA fragmentation levels (using the terminal deoxynucleotidyl transferase dUTP nick end labeling (TUNEL) method) and the expression levels of proapoptotic BAX, antiapoptotic Bcl-2, and pro-apoptotic cleaved caspase-3 in lung tissue 24 h after normal saline or endotoxin administration. Figure 8 illustrates the data of lung apoptosis, as measured by TUNEL assay (Figure 8a) and the expression levels of BAX (Figure 8b), Bcl-2 (Figure 8b), and cleaved caspase-3 (Figure 8b) in lung tissue. Our data demonstrate low TUNEL-positive cell counts in the Sham, the KCF, and the SEM groups, whereas the TUNEL-positive cell count in the Endo group was significantly higher than the Sham group (*p* < 0.001). The TUNEL-positive cell counts in the EKCF and ESEM groups were comparable (*p* = 0.99) and both were significantly lower than that of the Endo group (both *p* < 0.001). Of note, the trends of BAX and cleaved caspase-3 expressions and the BAX/Bcl-2 ratio paralleled that of the TUNEL-positive cell count data. Moreover, the expression levels of Bcl-2 in the Sham, the KCF, and the SEM groups were high, whereas the expression level of Bcl-2 in the Endo group was significantly lower than that of the Sham group (*p* = 0.012). In addition, the expression levels of Bcl-2 in the EKCF and the ESEM groups were comparable (*p* = 0.084) and both were significantly higher than the Endo group (*p* < 0.001 and =0.024). These data demonstrate the comparable effects of the KCF18 and SEM18 peptides on alleviating endotoxemia-induced apoptosis in lung tissues in mice.

## 3. Discussion

Data from the present study, in concert with those from a previous report [19], confirm that endotoxemia induced by Gram (-) endotoxin i.p. administration can cause significant systemic inflammation within 24 h after administration, as our data demonstrate significant upregulation of the early response inflammatory cytokines TNF-α, IL-1β, and IL-6 in plasma. Endotoxemia induced by Gram (-) endotoxin i.p. administration can also cause significant lung injury in mice within 24 h after administration, as our data demonstrate significant lung function alterations (including decreases in tidal volume, minute ventilation, and dynamic compliance; and by contrast, increases in airway resistance and end expiration work) and lung histological alterations (including increases in injury scores, leukocyte infiltration, vascular permeability, and tissue water content) in endotoxin-treated mice. Data from the present study further demonstrate that endotoxemia induced by Gram (-) endotoxin i.p. administration can cause inflammation (macrophage activation and cytokine upregulations), oxidative stress, and trigger the cell death processes of necroptosis, pyroptosis, and apoptosis in mouse lung tissue. Although one may argue that this monomicrobial sepsis model of endotoxemia cannot fully represent sepsis conditions observed in clinical situations [24], data from the present study provide clear evidence to confirm that this monomicrobial sepsis model of endotoxemia can readily induce significant lung injury within 24 h after sepsis induction. These data highlight the feasibility of this monomicrobial sepsis model of endotoxemia to be employed for the study of sepsis, especially for preclinical studies of lung injury induced by sepsis.

The KCF18 peptide possesses structural similarity to the binding domains of TNF-α, IL-1β, and IL-6 to the cognate receptors TNFR1, IL-1R, and IL-6R [21]. The KCF18 peptide can achieve triple cytokine (TNF-α, IL-1β, and IL-6) inhibition, by acting as a binding decoy to simultaneously inhibit respective receptor-ligand interactions [21]. The SEM18 peptide possesses structural similarity to the binding domain of TNF-α to TNFR1 [21]. The SEM18 peptide thus can achieve TNF-α inhibition, also through acting as a binding decoy to inhibit TNF-α/TNFR1 binding [21]. To facilitate investigations, this study employed these two peptides to achieve triple versus single cytokine inhibition. Data from this study, as with previous reports [21,22], demonstrate that the KCF18 peptide can achieve triple cytokine inhibition by limiting ligand-receptor interactions in lung tissue. Data from the study, also consistent with those from the previous reports [21,23], demonstrate that the SEM18 peptide can achieve TNF-α inhibition and inhibit endotoxin-induced TNF-α/TNFR1 binding in lung tissue. Data from the present study further demonstrate that the levels of cytokine upregulation in plasma and lung injury, inflammation, oxidation, necroptosis, pyroptosis, and apoptosis in mice receiving endotoxin plus the KCF18 or SEM18 peptide were significantly lower than those in mice receiving endotoxin alone. However, the peptides themselves did not differ in cytokine upregulation in plasma or injury, inflammation, oxidation, necroptosis, pyroptosis, and apoptosis. These data confirm our hypothesis that blocking TNF-α, IL-1β, and IL-6 (mediated by the KCF18 peptide) and blocking only TNF-α (mediated by the SEM18 peptide) can achieve similar effects on mitigating endotoxemia-induced systemic inflammation and lung injury in mice.

The effects of the SEM18 peptide on inhibiting TNF-α and blocking TNF-α/TNFR1 binding are confirmed in the present study. As TNF-α can modulate endotoxin-induced upregulation of IL-1β and IL-6 [8,13,16], we thus conjectured that the SEM18 peptide can decrease the endotoxemia-induced upregulation of IL-1β and IL-6 in plasma and lung tissues as well as the endotoxemia-induced bindings of IL-1β/IL-1R and IL-6/IL-6R in lung tissues. This concept is partially confirmed by the present study. Our data illustrate lower plasma and pulmonary IL-1β concentrations and lower level of IL-1β/IL-1R binding in lung tissues in mice receiving the SEM18 peptide plus endotoxin comparing to those in mice receiving endotoxin alone. Our data also demonstrate lower plasma IL-6 concentration in mice receiving the SEM18 peptide plus endotoxin comparing to that in mice receiving endotoxin alone. However, our data show that the pulmonary IL-6 concentration and the level of IL-6/IL-6R binding in lung tissues in mice receiving the SEM18 peptide plus endotoxin and those in mice receiving endotoxin alone were not significantly different. These data illustrate that TNF-α inhibition (mediated by the SEM18 peptide) did not exert significant effects on modulating endotoxemia-induced IL-6 upregulation and IL-6/IL-6R binding in mouse lung tissues. In line with this notion, it is thus reasonable to observe that the expression levels of Akt (namely the downstream pathway of IL-6) [18] in lung tissues in mice receiving the SEM18 peptide plus endotoxin and those in mice receiving endotoxin alone were not significantly different. Collectively, these data display that the SEM18 peptide significantly inhibited TNF-α and IL-1β, but not IL-6, in endotoxemia-treated mouse lung tissues.

Data from the present study, as above-described, show that the KCF18 peptide can achieve significant inhibitions of TNF-α, IL-1β, and IL-6 in endotoxemia mouse lung tissues, whereas the SEM18 peptide can achieve significant inhibitions of TNF-α and IL-1β, but not IL-6, in endotoxemia mouse lung tissues. However, with their differential effects on inhibiting IL-6, the present study illustrates that the KCF18 and SEM18 peptides can achieve similar effects on mitigating endotoxemia-induced systemic inflammation and lung injury in mice. These data support the concept that TNF-α, IL-1β, and IL-6 do not play equally important roles in mediating the development of lung injury in the early phase of endotoxemia, and the role of IL-6 in this regard should be supportive. Moreover, our data show that the SEM18 peptide can also inhibit IL-1β expression. As the SEM18 peptide can inhibit TNF-α and TNF-α modulates IL-1β expression [8,12], it is thus reasonable to observe the above-described effects of the SEM18 peptide on inhibiting IL-1β. Collectively, these data support the concept that TNF-α should be the major cytokine in mediating the development of lung injury in the early phase of endotoxemia.

Moreover, our data in concert with those previously reported [19,22,23], demonstrate that endotoxemia-induced lung injury also involves the cell death processes of necroptosis, pyroptosis, and apoptosis. Upon binding with its cognate receptor, TNFR1, the effects of TNF-α in triggering necroptosis, pyroptosis, and apoptosis are established [8,9,10,11,12]. As both peptides KCF18 and SEM18 can achieve similar effects on inhibiting endotoxemia-induced TNF-α/TNFR1 binding in mouse lung tissues, it is reasonable to observe our data that the levels of necroptosis, pyroptosis, and apoptosis in lung tissues in mice receiving the KCF18 peptide plus endotoxin and in mice receiving the SEM18 peptide plus endotoxin were comparable and significantly lower than those in mice receiving endotoxin alone. These data illustrate the involvement of necroptosis, pyroptosis, and apoptosis in mediating the development of endotoxemia-induced organ injury and highlight the therapeutic potentials of blocking TNF-α/TNFR1 binding against lung injury in the early phase of endotoxemia. 

This study employed peptide-based pharmacological approaches. Using the molecular docking simulation technique [25], the co-author Hsu developed two cytokine-capturing peptides (namely the KCF18 and SEM18 peptides) that can achieve triple versus single cytokine inhibition [21]. With the use of these two peptides, this study managed to uncover similar protection with both peptides in the early phase of endotoxemia. Using the simulation technique, the ideal binding sites for cytokine (or cytokines) to the cognate receptor(s) can be determined and the structure of the cytokine capturing peptide can then be designed and synthesized based on the simulation results [25]. Moreover, with the simulation technique, structure modification of the peptide can also be done in an efficient and timely manner. Peptides thus can be used as effective tools for pharmacological approaches to evaluate anticipated anti-inflammatory/immune modulators for therapy, as demonstrated by this study.

The crucial role of TNF-α in sepsis is well-established, as abundant data in this regard have been previously reported [1,2,5,6,7,8,9,10,11,12]. Several anti-TNF-α agents, e.g., monoclonal anti-TNF-α antibody, have been developed and their therapeutic effects on improving survival in sepsis patients have also been reported [26,27,28]. Similar to TNF-α, the crucial roles of IL-1β and IL-6 in sepsis are also established [1,2,5,6,7]. Several anti-IL-1β and anti-IL-6 agents have also been developed and clinical data also demonstrate their beneficial effects against sepsis [29,30]. However, data regarding the therapeutic effects of triple cytokine (TNF-α, IL-1β, and IL-6) inhibitions versus single cytokine (TNF-α) inhibition against sepsis remain lacking. As above-mentioned, this study employed a monomicrobial sepsis model of endotoxemia to facilitate investigation. With the novel peptide KCF18, this study managed to achieve simultaneous inhibitions of TNF-α, IL-1β, and IL-6 and thus can compare the therapeutic effects of triple versus single cytokine inhibitions against endotoxemia. Data from the present study, as above-described, illustrate that triple cytokine (TNF-α, IL-1β, and IL-6) inhibitions and single cytokine (TNF-α) inhibition can achieve similar therapeutic effects against endotoxemia-induced systemic inflammation and lung injury in the early phase of endotoxemia. These data support the concept that blocking TNF-α may be a more proximal intervention against endotoxemia, especially in the early phase of endotoxemia.

This study provides clear evidence to demonstrate the crucial role of TNF-α in mediating the development of systemic inflammation and lung injury in the early phase of endotoxemia as well as the feasibility and effectiveness of employing the peptide-based pharmacological approaches as research tools. However, certain study limitations do exist and need to be addressed. Firstly, this study focused on the early phase of endotoxemia; the roles of the investigated cytokines in the later phase of endotoxemia remain to be elucidated. Secondly, this study employed a monomicrobial sepsis model of endotoxemia [19] to facilitate investigations. The question of whether similar pictures can be observed with the other sepsis models, e.g., the polymicrobial sepsis model using cecal ligation and puncture [31], also remains to be elucidated. Thirdly, this study employed only one therapeutic regimen of the respective KCF18 and SEM18 peptides; the question of whether the therapeutic effects of the KCF18 peptide and/or the SEM18 peptide are dose-dependent remains unanswered. Fourthly, our data show that the SEM18 peptide did not exert significant effects on modulating IL-6 expression and IL-6/IL-6R binding. The mechanisms remain to be elucidated. Fifthly, as above-mentioned that we previously conducted two studies to confirm the therapeutic effects of the KCF18 and SEM18 peptides on mitigating endotoxemia-induced liver injury [22,23]. Data from those previous studies revealed that both KCF18 and SEM18 peptides did not exert significant effects on liver function and did not cause liver injury in mice receiving normal saline. In contrast, data from the present study demonstrate that both peptides can exert certain effects in lung function in mice receiving normal saline. Moreover, the KCF18 peptide may induce mild lung injury in mice receiving normal saline. These data indicate that the peptides employed in this study, especially the KCF18 peptide, may not be harmless. The mechanisms remain to be elucidated. Nevertheless, these data indicate that more studies and certain structural modifications of the peptides should be conducted to ensure the safety of the peptides before further clinical applications can be considered. Sixthly, data of the lung function tests show significant difference between the study groups (i.e., *p* values < 0.001). However, the difference, even though significant, is actually very small between the study groups. The clinical significance of these data thus needs validation before further conclusion can be drawn. Seventhly, data from the present study provide clear evidence to support the concept that these two peptides mainly act through inhibiting cytokine-receptor bindings to exert their effects against endotoxemia-induced acute lung injury in mice. However, endotoxemia-induced organ injury involves complicated pathways. It is possible that other pathways may also participate in mediating the demonstrated therapeutic effects of the SEM18 and KCF18 peptides in the present study. More studies are needed before further conclusion can be drawn on this issue. Eighthly, data of the present study are murine data. We performed a follow up human cell based in vitro experiment employing a human monocytic THP-1 cell line. THP-1 cells were stimulated, as we have previously reported [32]. The data demonstrate that these two peptides can exert comparable and significant effects on inhibiting NLRP3 upregulation (please refer to the Supplemental Figure S1). These data support the concept that these two peptides may exert similar effects in human and mice. Nevertheless, human data are needed before clinical application can be considered.

**Figure 9:**
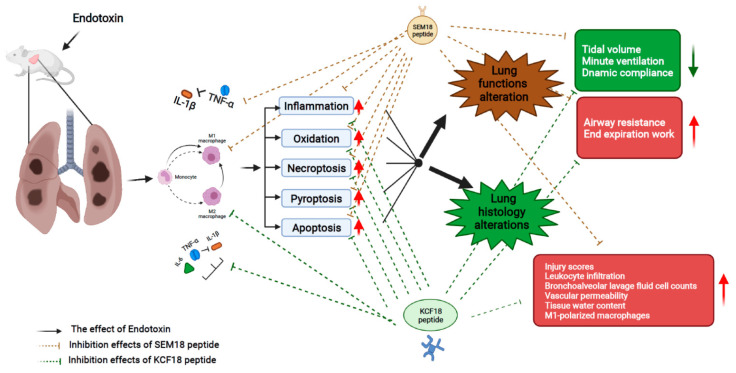
The diagram illustrating the effects and mechanisms of inhibiting the early response cytokines tumor necrosis factor-α (TNF-α), interleukin-1β (IL-1β) and/or interleukin-6 (IL-6) (as achieved by the novel peptides KCF18 and SEM18) on inhibiting endotoxin-induced lung injury in the early phase of endotoxemia (i.e., 24 h after endotoxemia induction) in mice.

The effects and mechanisms, as above described, are summarized in Figure 9.

## 4. Materials and Methods

### 4.1. Study Animals

This study used adult male mice (BALB/cJ mice, 7–8 weeks old), purchased from Taiwan’s National Laboratory Animal Center (Taipei, Taiwan). Care of the mice was handled according to the US National Institutes of Health guidelines. Mice were kept in a setting of 12-h light/12-h dark cycle with free water access and maintained with a regular diet for laboratory mice.

### 4.2. Designs and Syntheses of Peptides

Designs of the KCF18 and SEM18 peptides (molecular weight: 2195.92 and 2161.54 Da, respectively) were conducted using the “molecular docking simulation technique” (ZDOCK, BIOVIA Discovery Studio 3.5; BIOVIA, San Diego, CA, USA) [25], as per the previous report [21]. The KCF18 and SEM18 peptides were manufactured (Mission Biotech, Taipei, Taiwan) and the assaying data of high-performance liquid chromatography revealed that the purities of both peptides were approximate 95%.

### 4.3. Monomicrobial Sepsis Model of Endotoxemia and Peptide Therapy

As aforementioned, this study used a Gram (-) endotoxin (*E. coli* 0127: B8 endotoxin; Sigma-Aldrich, St. Louis, MO, USA) i.p. administration model to engender monomicrobial sepsis in mice [19]. Three groups of mice were arbitrarily assigned to be given endotoxin (15 mg/kg, i.p.), endotoxin plus the KCF18 peptide, or endotoxin plus the SEM18 peptide (identified as the Endo, EKCF and ESEM group, respectively). Another 3 groups of mice were arbitrarily assigned to be given normal saline (0.5 mL per mice, i.p.), normal saline plus the KCF18 peptide, or normal saline plus the SEM18 peptide (identified as the Sham, KCF, and SEM group, respectively) and used as the individual control of the groups with endotoxin. Administrations of peptides, KCF18 and SEM18, were carried out, as per our previous reports [22,23]. The KCF18 peptide (0.6 mg/kg, i.p.) was injected at 2 h after endotoxin or normal saline, followed by three auxiliary doses (0.3 mg/kg, every 2 h) [22]. Similarly, the SEM18 peptide (0.3 mg/kg, i.p.) was injected at 2 h after endotoxin or normal saline, followed by three auxiliary doses (0.15 mg/kg, every 2 h) [23].

All mice were maintained as above-mentioned and intensively monitored. At 24 h after endotoxin or normal saline injection, the mice were anesthetized with a mixture of zoletil and xylazine (40/10 mg/kg, i.p.) to facilitate lung function assay, BALF collection, blood sample collection, or vascular permeability assay. Then, mice were decapitated for euthanasia followed by lung tissue collection.

### 4.4. Lung function Assay

The anesthetized mice received a tracheostomy and a 20G catheter (B. Braun; Melsungen, Germany) was inserted as the tracheostomy tube. The tube was then connected to a computerized small animal ventilator (Finepoint; Buxco Electronic, Wilmington, NC, USA). The mechanical ventilation was set at a ventilation rate of 150 breaths/min and a tidal volume of 0.2 mL. The airway resistance and dynamic compliance were recorded using a FinePointe™ RC System (Buxco Research Systems; New Brighton, MN, USA). Controlled respiration rate, tidal volume, minute volume, dynamic compliance, resistance and end expiration work was obtained, as per a previous report [33].

### 4.5. BALF Collection and Assay

A set of anesthetized mice from each group received 5 times lavage with 1 mL sterile normal saline via the tracheostomy tube. The BALF samples were then collected, as per our previous report [34]. An aliquot of BALF was diluted 1:1 with trypan blue dye (Sigma-Aldrich), and the total cell number in BALF was measured.

### 4.6. Lung Tissue and Plasma Samples Harvesting and W/D Ratio Assay

The anesthetized mice received a midline laparotomy and sternotomy to expose the abdominal aorta and the lungs. The blood samples were harvested via aortal puncture. The blood samples were then centrifuged and the supernatants (i.e., the plasma) were collected and stored (−20 °C) for later analysis. After euthanasia, the trachea was ligated and the left and right lungs were freshly dissected and removed. The freshly harvested lung tissues were divided and collected. Half of the collected lung tissue samples were snap frozen in liquid nitrogen and stored (−80 °C) for later analysis. The other half of the collected lung tissue samples were used for later W/D ratio assay. In addition, a set of mice in each group received 10% formalin solution (Sigma-Aldrich) perfusion via the tracheostomy tube and the lung tissues were then removed and harvested for histologic analysis.

W/D ratio assay was conducted to determine lung water content (i.e., a lung injury marker), as per our previous report [34]. In brief, the freshly harvested lung tissue samples were weighed, placed in the oven (80 °C) for 24 h, and then weighed again. The W/D ratios of the lung tissue samples were then calculated.

### 4.7. PLA Method for Cytokine/Receptor Binding Analysis

The PLA method [35] was conducted to analyze the binding condition of cytokine to the cognate receptor in lung tissues, using a PLA kit (DuoLink Mouse Rabbit in situ PLA kit; Sigma-Aldrich). In brief, lung tissue sections were blocked and then incubated with the primary antibodies (i.e., TNF-α and TNFR1, IL-1β and IL-1R, or IL-6 and IL-6R; all from Abcam, Cambridge, UK), followed by incubation with the secondary antibody (i.e., the oligonucleotide-labeled PLA probe). Nuclei were stained with 4′,6-diamidino-2-phenylindole (DAPI; Sigma-Aldrich). Images of all tissue sections were observed (DeltaVision Elite microscope; GE Healthcare, Marlborough, MA, USA) and scanned. The PLA signal intensities of the scanned images were measured using the image processing software ImageJ (a free software from https://imagej.nih.gov/ij/, accessed on 11 July 2018).

### 4.8. Histological Analysis for Lung Injury Assay

The formalin-infused lung tissues were placed in paraffin wax, followed by sequential sectioning, and then stained with hematoxylin and eosin, as per our previous report [34]. Lung injury was evaluated according to the histological characteristics, including alveolar wall edema, hemorrhage, vascular congestion, and polymorphonuclear leukocytes (PMN) infiltration, using a light microscope. Each of the histological characteristics was further rated, based on a scale of 0 to 5 (normal to severe). The sum (i.e., the lung injury score) was then calculated to determine lung injury levels [34].

### 4.9. EBD Extravasation Assay for Vascular Permeability Analysis

EBD extravasation assay [36] was conducted to analyze vascular permeability in the lungs. In brief, a set of anesthetized mice from each group received intravenous (i.v.) injection of EBD (2 mL/kg, 2% solution in normal saline; Sigma-Aldrich) at 23 h after endotoxin or normal saline injection, followed by thorough perfusion with normal saline (i.v.), starting at 1 h after EBD injection, to eliminate residual EBD. After euthanasia, the lung tissue samples were harvested, weighed, blended in 50% trichloroacetic acid (1:3 volume ratios; Sigma-Aldrich), and then centrifuged. The collected supernatants were analyzed with spectroscopy (absorbance: 620 nm) to measure the concentrations of EBD.

### 4.10. ELISA Method for Cytokine Analysis

Concentrations of cytokines in plasma and the lung tissue samples were measured using the ELISA method, as per our previous report [34]. The snap frozen lung tissue samples were blended in extraction buffer (Enzo Life Science, Farmingdale, NY, USA) and centrifuged to aid supernatants collection. Then, the concentrations of TNF-α, IL-1β, and IL-6 in the samples of plasma and supernatants were measured using ELISA kits (TNF-α, IL-1β, and IL-6 ELISA kit; Enzo).

### 4.11. Immunohistochemistry Staining Assay for Inflammation, Oxidation, Necroptosis, and Pyroptosis Analyses

The immunohistochemical assay, as per our previous reports [22,23], was conducted to analyze the following crucial mechanisms, including inflammation, oxidation, and cell death processes of necroptosis and pyroptosis. In brief, lung tissue paraffin sections were prepared and then conjugated with one of the following primary antibodies (all from Abcam), including anti-iNOS (a marker for M1 phase macrophage polarization) [30], anti-CD206 (a marker for M2 phase macrophage polarization) [37], anti-MDA (a marker for lipid peroxidation) [38], anti-pMLKL (necroptosis-related protein) [39], and anti-NLRP3 (pyroptosis-related protein) [15]. All sections were observed (TissueGnostics Axio Observer Z1 microscope; TissueGnostics GmbH, Vienna, Austria) and scanned. The scanned images were then analyzed with the image processing software Image J.

### 4.12. TUNEL Method for Apoptosis Analysis

The TUNEL method, as per our previous reports [22,23], was conducted to analyze the apoptosis status in lung tissues through measuring DNA fragmentation (i.e., the key characteristic of apoptosis) [40]. A commercial kit (the in-situ cell death detection kit; Roche, Indianapolis, IN, USA) was used and the procedures for apoptotic cell staining in the lung tissue samples were conducted, as per the manufacturer’s protocols. Nuclei were stained with DAPI (Sigma-Aldrich). All sections were then observed, scanned and analyzed, as above described. The TUNEL-positive cell counts in five arbitrarily selected fields (0.25 mm^2^) of each lung tissue section were measured. The mean TUNEL-positive cell counts were then calculated to determine the lung apoptosis status in each group.

### 4.13. Immunoblotting Assay for Akt, Necroptosis, Pyroptosis, and Apoptosis Analyses

The immunoblotting assay, as per our previous reports [22,23], was conducted to analyze the following crucial mechanisms, including expression of Akt as well as cell death processes of necroptosis, pyroptosis, and apoptosis. In brief, the snap-frozen lung tissue samples were blended in lysis buffer (Abcam) and centrifuged to aid supernatants collection and protein extraction. After determining the protein concentrations, equal amounts of proteins (100 μg) from each sample were loaded into the wells of the gel (12.5%; Abcam) and then separated by electrophoresis, followed by transfer to nitrocellulose membranes (Bio-Rad Laboratories, Hercules, CA, USA). The membranes were then conjugated with one of the following primary antibodies, including anti-phosphorylated Akt (anti-pAkt, Cell Signaling Technology, Danvers, MA USA), anti-Akt (Cell Signaling), anti-cleaved caspase-8 (the necroptosis-related protein, Abcam, Cambridge, UK), anti-caspase-8 (Abcam), anti-cleaved caspase-1 (the pyroptosis-related protein, Abcam), anti-caspase-1 (Abcam), anti-BAX (the proapoptotic protein, Abcam,), anti-Bcl-2 (the antiapoptotic protein, Cell Signaling), anti-cleaved caspase-3 (the proapoptotic protein, Abcam), or anti-actin (the internal standard; Sigma-Aldrich). The chemiluminescence method was then conducted to detect the bound antibody (ECL Plus kit; Amersham, Buckinghamshire, UK). The membranes were then scanned and the density of each protein band was measured using the densitometry method (ImageJ). Moreover, the BAX/Bcl-2 ratio was also calculated to measure the apoptosis status in each group [20,21].

### 4.14. Statistical Analysis

The data were calculated and illustrated as mean ± standard deviations. To analyze the between-group differences, as per our previous reports [20,21], one-way analysis of variance and Tukey’s test for post hoc pairwise comparisons were conducted. A *p*-value of less than 0.05 was identified as statistically significant. This study used the statistical software SPSS v21.0 (SPSS, Somers, NY, USA) for data analysis.

## 5. Conclusions

Data from this study demonstrate that TNF-α is the major cytokine in mediating the development of systemic inflammation and lung injury in the early phase of endotoxemia. Moreover, peptides can serve as effective tools for pharmacological research.

## Data Availability

Data is contained within the article and Supplementary Material.

## Supplementary Materials

The following supporting information can be downloaded at: https://www.mdpi.com/article/10.3390/ph15030287/s1, Figure S1: Effect of peptides KCF18 and SEM18 on modulating NLR family pyrin domain containing 3 (NLRP3) transcriptional expression in the human monocytic *cell* line THP-1 cells stimulated with endotoxin as well as the uncropped original representative gel images of immunoblotting assay.
