# Supplementary material for: Tumor Necrosis Factor-α Mediates Lung Injury in the Early Phase of Endotoxemia
Source: Pharmaceuticals (Basel). 2022 Feb 25;15(3):287. doi: 10.3390/ph15030287 (PMC8953981; doi:10.3390/ph15030287)
Supplement: Supplementary file 1 [file pharmaceuticals-15-00287-s001.zip › pharmaceuticals-1617156-supplementary.pdf]

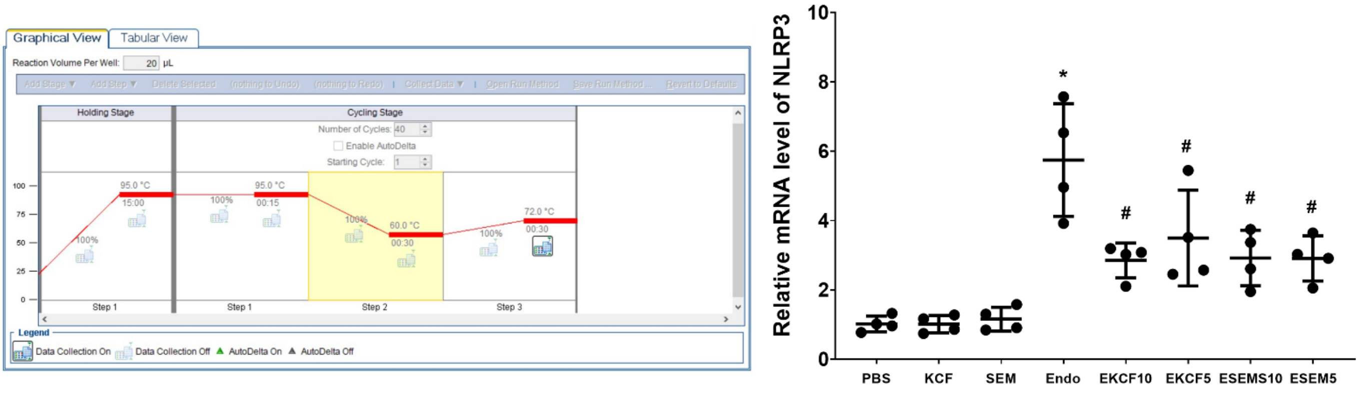

Supplemental Figure S1. Effect of peptides KCF18 and SEM18 on modulating NLR family pyrin domain containing 3 (NLRP3) transcriptional expression in the human monocytic cell line THP-1 cells stimulated with endotoxin. NLRP3 mRNA levels in the cell lysates of the THP-1 cells were measured using real-time polymerase chain reaction (PCR). For cell activation, THP-1 cells were incubated with phorbol 12-myristate 13-acetate (100 nM) for 72 hours. After activation, the THP-1 cells (1×10^6 cells per well) were stimulated with endotoxin (1 µg/mL) or phosphate buffered saline (PBS). At 2 hours after LPS or PBS stimulation, KCF18 peptide (5 or 10 µM)) or SEM18 peptide (5 or 10 µM) were administered to THP-1 cells. At 4 hours after endotoxin or PBS administration, THP-1 cells were harvested and the cell lysates were collected. With the following primers: NLRP3, 5′-CTACACACGACTGCGTCTCA TCAA-3′ and 3′-GGGTCAAACAGCAACTCCATCTTA-5′ and GAPDH, 5′-AGAAGGCTGGGGCTCATTTG-3′, and 3′-AGGGGCCATCCACAGTCTTC-5′, real-time PCR was performed according to our previous report (Chang, Y.Y.; et al. *Inflammation*. **2020**; 43: 1375) to measure NLRP3 mRNA levels in cell lysates. The levels of NLRP3 mRNA were normalized to the levels of GAPDH mRNA in each group and reported as the ratio to the PBS group. PBS: the PBS group, KCF: the KCF18 peptide 10 µM group. SEM: the SEM18 peptide 10 µM group. Endo: the endotoxin group. EKCF10: the endotoxin plus KCF18 peptide 10 µM group. EKCF5: the endotoxin plus KCF18 peptide 5 µM group. ESEM10: the endotoxin plus SEM18 peptide 10 µM group. ESEM5: the endotoxin plus SEM18 peptide 5 µM group. Data are mean ± standard deviations (n = 4 in each group). *$p < 0.05$ versus the PBS group. #$p < 0.05$ versus the Endo group.

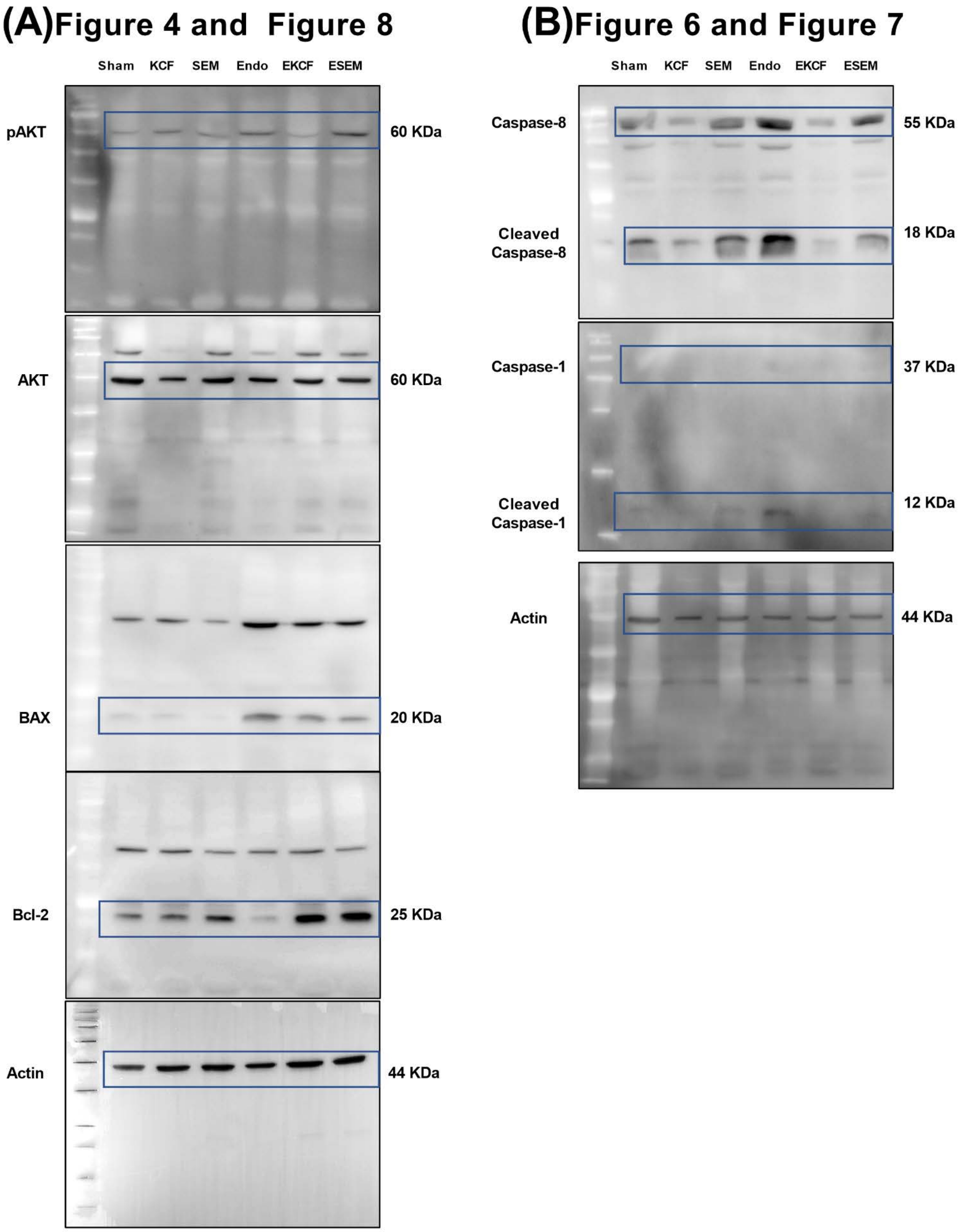

The uncropped original representative gel images of immunoblotting assay. Sham: the normal saline group. KCF: the normal saline plus the KCF18 peptide group. SEM: the normal saline plus the SEM18 peptide group. Endo: the endotoxin group. EKCF: the endotoxin plus the KCF18 peptide group. ESEM: the endotoxin plus the SEM18 peptide group.
